# Physiological Variables that Contribute to Aerobic Fitness in Boys during Early Adolescence in the Context of Basketball Training and the Maturity Level

**DOI:** 10.5114/jhk/159627

**Published:** 2023-01-20

**Authors:** Eligijus Mačinskas, Loreta Stasiulė, Kęstutis Pužas, Arvydas Stasiulis

**Affiliations:** 1 Department of Health Promotion and Rehabilitation, Lithuanian Sports University, Kaunas, Lithuania.

**Keywords:** team sports, VO_2peak_, cardiac output, stroke volume, respiratory function

## Abstract

The aim of this study was to assess physiological variables that contribute to aerobic fitness in respect to basketball training and the maturity level in adolescent boys. Our subjects were 28 basketball-trained and 22 control-group boys (average age: 11.83 ± 0.43 years). An incremental treadmill running test to exhaustion was performed twice with a 1-year interval between the sessions to determine the following peak aerobic fitness variables: oxygen uptake, stroke volume, cardiac output, minute ventilation, and others. Maturity offset was used to evaluate the maturity level. The basketball-trained group exhibited a higher peak ratio-scaled oxygen uptake (1^st^ session: 50.55 ± 6.21 and 46.57 ± 5.68 ml/kg/min in basketball and control-group boys, respectively, p = 0.024; 2^nd^ session: 54.50 ± 6.50 and 45.33 ± 5.99 ml/kg/min, respectively, p < 0.001) during both testing sessions. During the 2^nd^ session, the basketball-trained group also showed a significantly higher peak arteriovenous oxygen difference (basketball-trained boys: 14.02 ± 2.17 ml/100 ml; control-group boys: 12.52 ± 2.49 ml/100 ml; p = 0.027) and peak minute ventilation (basketball-trained boys: 96.08 ± 21.71 l/min; control-group boys: 83.14 ± 17.85 l/min; p = 0.028). The maturity level among the basketball-trained boys was correlated with peak variables: oxygen uptake, stroke volume, cardiac output, and minute ventilation, but not with the ratio-scaled oxygen uptake. In conclusion, basketball training at a young age among boys improved aerobic fitness compared with sedentary boys. More mature basketball players were not superior to their less mature peers regarding aerobic fitness after adjusting for body dimensions.

## Introduction

Aerobic fitness (AF) can be considered one of the most important body physical abilities, both in the general population and among athletes of any age (Armstrong et al., 2011). This body performance ability is usually tested during an incremental intensity exercise with pulmonary gas exchange analysis by identifying peak or maximal oxygen uptake (VO_2peak_ or VO_2max_) levels. In children, AF increases with growth and maturation (Armstrong and Welsman, 2019c). Moreover, AF can also change as the result of adaptation to exercise training (Armstrong et al., 2011; Vamvakoudis et al., 2007).

In a previous study, Armstrong et al. (2011) reviewed the effect of sport on AF in youths and stated that young athletes had higher VO_2peak_ than their untrained peers. In addition, Vamvakoudis et al. (2007) observed superior VO_2peak_ in basketball-trained (BT) 11.5-year-old boys compared with their untrained control-group (CG) peers at the initial testing and during the 2-year follow-up. In contrast, one recent study argued that children’s training-induced changes in VO_2peak_ are typically well below 10%, and that improvements in VO_2peak_ after a similar training stimulus are much smaller than those observed in adults (Dotan, 2017). In their longitudinal study, Mirwald et al. (1981) did not observe differences in VO_2peak_ between active and sedentary children until adolescence. Therefore, the effect of sport participation on VO_2peak_ has not been fully elucidated (Armstrong and van Mechelen, 2017).

Based on earlier studies, AF can be defined as the body’s ability to extract oxygen, deliver it to the working muscles, and then utilize it to generate energy (Armstrong et al., 2011). VO_2peak_ is the most commonly known and widely used physiological variable to characterize the individual level of AF. Conversely, pulmonary ventilation does not limit the peak VO2 in healthy children (Armstrong and van Mechelen, 2017) and, according to the Fick’s equation, VO2 is the product of the difference in arteriovenous oxygen concentration (a-vO2 diff) and cardiac output (Q). Therefore, the variables mentioned above, together with the peak heart rate (HRpeak) and peak stroke volume (SVpeak), are also of interest for providing a comprehensive view of the cardiorespiratory system. To the best of our knowledge, no other study has evaluated the effect of basketball training on all of the corresponding variables mentioned above in children.

Sport has become not only a social activity, entertainment, or a way of competing with others, but also a critical component of a person’s health and well-being (Bize et al., 2007). Therefore, exercising is widespread in different population groups. In particular, basketball is considered as one of the most popular sports worldwide. It is also one of the most physically demanding team sports; thus, the sufficient AF of players is paramount to their ability to recover and maintain a high-intensity activity level during the entire game, which can now occasionally span more than 90 min (Ziv and Lidor, 2009). Many activities in basketball are considered anaerobic because they are performed at maximal or submaximal intensities. Conversely, activities like sprinting and jumping are interspersed with periods of slow running, walking, or resting during the game-stops, which suggests that the game is similar to high-intensity interval training (HIIT) (Sekine et al., 2019). HIIT is a very effective and time-efficient way to improve AF levels in adults (Gist et al., 2014). Hence, improvements in AF are plausible as a consequence of long-term participation in basketball practice.

It is also known that on-court success in basketball during childhood and adolescence is correlated with the physical abilities of players, including endurance (Torres-Unda et al., 2013). More mature young athletes, including judokas, soccer, and basketball players, are usually more successful during their teen years because of their increased physicality and body dimensions (Duarte et al., 2019; Giudicelli et al., 2021; Guimarães et al., 2019). VO_2peak_ increases with age because of morphological changes mainly in body mass and fat-free mass (FFM) (Armstrong and Welsman, 2019a). Nevertheless, greater clarity is needed regarding the maturity-related differences in peak cardiorespiratory variables in children.

We hypothesized that long-term basketball training would have a positive impact on peak cardiorespiratory variables (VO_2peak_, Q_peak_, and SV_peak_). Second, we predicted that maturity-induced morphological changes would also positively affect VO_2peak_, Q_peak_, and SV_peak_ among young basketball players. Therefore, the objective of this study was to assess the AF profile of pre-trained young basketball players in comparison to the sedentary CG boys during an incremental treadmill run to exhaustion. Subsequently, a 1-year follow-up was applied to check for further differences in AF caused by the continuous basketball training process.

## Methods

### Participants

A sample of 50 boys (BT: *n* = 28, CG: *n* = 22; age during the first session: 11.83 ± 0.43 years) participated in both testing sessions. The BT group consisted of boys from two local basketball schools, whereas CG boys were from a local public school. All participants in this study were volunteers. The demographics of the subjects are presented in Table 1.

Both groups had similar inclusion criteria: 1) no chronic diseases, 2) approval to participate in HIIT, 3) voluntary participation, and 4) parental approval to participate. In addition to this, BT boys were actively attending basketball practices (3+ times/week), whereas CG boys had no more than two leisure-time practice sessions of different types per week.

Written informed consent was obtained from all participants and their parents or guardians before conducting the tests. The study was approved by the Lithuanian National Committee for Bioethics (date: 30.05.2018; Nr: BE-2-28) and adhered to the guidelines of the Declaration of Helsinki.

### Measures

Each session started by measuring the height of participants (vertical standing height and height while sitting on a chair with a height of 43 cm) using a vertical stadiometer (Leicester HM-250P, Marsden, Rotherham, UK). Subsequently, a total body composition analyzer (TBF-300A, Tanita, Tokyo, Japan) was used to measure body mass and FFM. Then, participants were asked to fill out a short questionnaire regarding their high-intensity physical activity time (including physical education lessons at school, leisure-time practice sessions, playing with friends, etc.) during a standard week. The use of a questionnaire in determining the physical activity level was chosen because of the relatively easy and cheap methodology in comparison with electronical monitoring. Furthermore, an incremental run-to-exhaustion test was performed on a treadmill (Lode, Lode BV Medical Technology, Groningen, The Netherlands) to evaluate the cardiorespiratory function of the subjects. After a short warm-up (slow walking for 3 min at 3 km/h), participants ran at an initial constant speed of 6 km/h for 4 min. Subsequently, the speed increased at a fixed rate of 0.1 km/h every 6 s. Participants continued the test until exhaustion or because they could not maintain the developed speed. The incline of the treadmill belt was set at 1% during the whole run. Members of the research team verbally encouraged participants to continue running during the last stage of the test.

A portable gas analyzer (Oxyconn Mobile, Jaeger Inc., Höchberg, Germany) was used to continuously record respiratory variables: VO_2_, VO_2kg_, respiratory rate (RR), pulmonary ventilation (VE), tidal volume (TV), and respiratory exchange ratio. These variables were measured on a breath-by-breath basis, and they were recorded and presented live; the data were averaged every 5 s by software provided by the manufacturer. Before starting each test, the gas analyzer was calibrated according to the procedures described by the manufacturer using a sample gas mixture with a known concentration. Simultaneously, the HR was recorded using a heart rate monitor (Polar S-810, Polar Electro Oy, Kempele, Finland) that was placed on the chest of participants using an elastic belt. The contact point between the electrodes and the subjects’ skin was covered with a special gel, to enhance the quality of the electrical signal. A portable noninvasive bioimpedance cardio output device (Physioflow PF07 Enduro, Manatec Biomedical, Poissy, France) was used to record SV and Q during the incremental run. Before the test, each participant was prepared according to the manufacturers’ recommendations. Individual device calibration using age, body mass, stature, and resting blood pressure of each participants was performed. Cardio data were also recorded and presented as live averages every 5 s. The continuous change in a-vO2 diff was not directly measured; rather, it was calculated after the test using the Fick’s equation, as follows: a-vO2 diff = VO2/Q. Each peak value (VO_2peak_, HR_peak_, SV_peak_, Q_peak_, a-vO2 diff peak, VE_peak_, RR_peak_, and TV_peak_) was determined as the peak value attained during the incremental running test (data were averaged every 30 s).

A mathematical equation reported previously (Mirwald et al., 2002) was applied to calculate the maturity offset (OS). Age at peak height velocity (APHV) is considered to indicate the timing of the most rapid maturity-related body changes in adolescents, whereas OS indicates the number of years that are left until (negative result) or have passed since (positive result) the APHV. Therefore, a lower OS result implies a lower maturity level.

### Design and Procedures

The study consisted of two testing sessions that were performed at a 1-year interval. Testing was carried out in spring because in our country the end of the children’s basketball season occurs at this time of the year and boys from all teams are usually in their peak form. Each physical assessment of participants was conducted individually, one participant at a time. All tests were conducted at the university testing facility.

### Statistical Analysis

The Shapiro-Wilk test was used to verify the normality of data distribution, and the Levene’s test was used to check the homogeneity of variances between the groups. The confidence interval in all statistical calculations was set at 95%. The differences in peak cardiorespiratory variables between the groups during both testing sessions were evaluated separately using analysis of variance (ANOVA). Mixed ANOVA was used to compare the differences in the 1-year changes between the BT and CG subjects. The correlation between peak cardiorespiratory variables and the maturity level was evaluated using Pearson’s correlation coefficients. The IBM SPSS 26 (IBM Statistics for MacOS, Released 2019, Version 26.0, IBM Corp., Armonk, NY, USA) software was used for all statistical calculations.

## Results

Table 1 presents descriptive and anthropometric characteristics of BT and CG boys. The sample groups were considered homogeneous because most variables were similar between the participants during both testing sessions.

**Table 1:** Descriptive and anthropometric characteristics of basketball-trained and untrained control-group boys during both testing sessions.

	Basketball-trained boys (*n* = 28)				Control-group boys (*n* = 22)			
	1^st^ testing session		2^nd^ testing session		1^st^ testing session		2^nd^ testing session	
Characteristics	mean	SD	mean	SD	mean	SD	mean	SD
Age (years)	11.84	0.38	12.88^#^	0.40	11.81	0.49	12.88#	0.56
Height (cm)	158.4*	6.5	165.7^#^	7.3	154.3	7.6	162.4#	9.1
Body mass (kg)	44.09	7.44	50.31^#^	9.57	43.68	7.97	48.96#	8.76
FFM (kg)	38.79	5.41	44.37^#^	7.17	36.72	5.57	41.74#	6.72
APHV (years)	13.61	0.38	13.56	0.49	13.79	0.39	13.79	0.56
OS (years)	–1.73	0.46	–0.65^#^	0.54	–1.98	0.59	–0.92#	0.77
PAT (h/week)	8.07*	2.19	8.84*	2.40	4.01	1.62	4.05	1.83

FFM, fat-free mass; APHV, age at peak-height velocity; OS, maturity offset; PAT, high-intensity physical activity time; SD, standard deviation

* Significant difference (p < 0.05) between the groups on certain testing sessions

^#^ Significant difference (p < 0.05) within the group between the testing sessions

**Figure 1:**
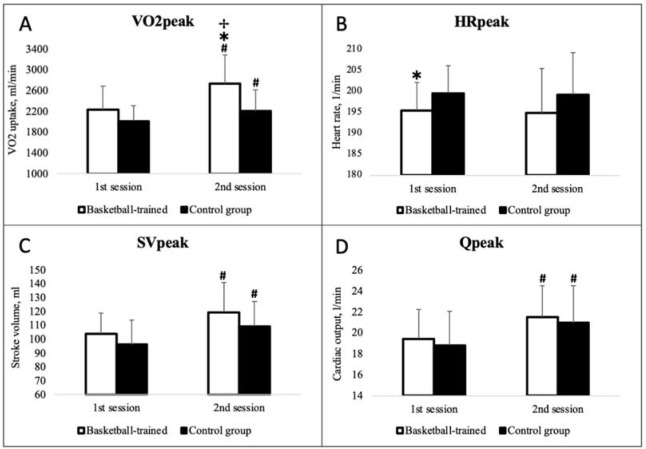
Peak cardiorespiratory variables of basketball-trained and untrained control-group boys during the two testing sessions. VO2: oxygen uptake; HR: heart rate; SV: systolic volume; Q: cardiac output. * - significant difference between-groups on certain testing session (p < 0.05) # - significant change within-groups between different sessions (p < 0.05) ✢ - significant factor (group) influence to between-sessions change (p < 0.05)

The most important peak cardiorespiratory variables were compared between the BT and CG boys (Figure 1). A significant within-group increase in the following variables was observed in BT boys between testing sessions: VO_2peak_ (1^st^ session: 2228.15 ± 453.99 ml/min; 2^nd^ session: 2731.58 ± 550.21 ml/min; *p* < 0.001; part A), SVpeak (1^st^: 103.87 ± 14.75 ml; 2^nd^: 119.22 ± 21.81 ml; *p* < 0.001; part C), and Q_peak_ (1^st^: 19.41 ± 2.80 l/min; 2^nd^: 21.51 ± 2.97 l/min; *p* < 0.001; part D). In CG boys, significant improvements were observed during the 1-year period in VO_2peak_ (1^st^: 2009.07 ± 302.89 ml/min; 2^nd^: 2202.05 ± 409.52 ml/min; *p* = 0.002; part A), SVpeak (1^st^: 96.23 ± 17.30 ml; 2^nd^: 109.23 ± 17.88 ml; *p* < 0.001; part C), and Qpeak (1^st^: 18.82 ± 3.21 l/min; 2^nd^: 20.95 ± 3.53 l/min; *p* < 0.001; part D).

During the 1^st^ testing session, both groups showed similar peak aerobic capacity (*p* > 0.05), with the exception of CG boys, who reached higher HRpeak values (BT: 195.41 ± 6.72 1/min; UT: 199.55 ± 6.50 1/min; *p* = 0.033). Conversely, during the 2^nd^ session, we observed significantly better results of VO_2peak_ in the BT group (BT: 2731.58 ± 550.21 ml/min; UT: 2202.05 ± 409.52 ml/min; *p* < 0.001).

Additional peak cardiopulmonary variables are presented in Table 2. Within-group increases between the testing sessions in BT boys were observed in VO_2kgpeak_ (*p* = 0.007), VO_2ffmpeak_ (*p* = 0.009), VEpeak (*p* < 0.001), and TVpeak (*p* < 0.001). Similarly, VEpeak (*p* = 0.007) and TVpeak (*p* = 0.012) improvements were observed in CG boys. Table 2 also reports that, during the first testing session, BT boys had higher VO_2kgpeak_ (*p* = 0.024). Conversely, during the 2^nd^ session, we observed significantly better results for VO_2kgpeak_ (*p* < 0.001), VO_2ffmpeak_ (*p* < 0.001), a-vO2 diff peak (*p* = 0.027), and VE_peak_ (*p* = 0.028) in the BT group.

**Table 2:** Peak cardiorespiratory variables of basketball-trained and untrained control-group boys.

	Basketball-trained boys (*n*= 28)				Control-group boys (*n*= 22)			
	1^st^ testing session		2^nd^testing session		1^st^testing session		2^nd^testing session	
Peak parameters	Mean	SD	Mean	SD	Mean	SD	Mean	SD
VO_2kgpeak_ (ml/kg/min)	50.55*	6.21	54.50^#*✢^	6.50	46.57	5.68	45.33	5.99
VO_2ffmpeak_(ml/kg_ffm_/min)	57.28	7.45	61.50^#*✢^	7.15	55.03	5.98	53.04	6.93
a-vO2diff peak (ml/100 ml)	13.31	2.61	14.02*	2.17	12.05	1.77	12.52	2.49
VEpeak(l/min)	81.08	14.27	96.08^#*✢^	21.71	75.75	13.98	83.14#	17.85
RR_peak_(1/min)	62.92	10.36	61.56	9.37	63.51	8.62	60.86	9.77
TV_peak_(l)	1.49	0.25	1.72^#^	0.30	1.43	0.30	1.60^#^	0.41
RER_peak_	1.01	0.05	1.01	0.05	1.00	0.05	1.02	0.07

VO2, oxygen uptake; a-vO2 diff, arteriovenous blood oxygen concentration difference; VE, minute ventilation; RR, respiratory rate; TV, tidal volume; RER, respiratory exchange ratio * Significant difference (p < 0.05) between the groups on certain testing sessions # Significant difference (p < 0.05) within the group between testing sessions ✢ Significant factor (group) effect on between-sessions changes (p < 0.05)

Table 3 shows the correlations between peak cardiorespiratory variables and the maturity level (calculated as OS) in BT boys. VO_2kgpeak_ and RR_peak_ were not correlated with maturity. In contrast, VO_2peak_, SV_peak_, and VE_peak_ were correlated with maturity in the BT group during both testing sessions.

**Table 3:** Correlations between peak cardiorespiratory variables and the maturity level in basketball-trained boys.

	1^st^ session	2^nd^ session
VO_2peak_	0.508**	0.666**
VO_2kgpeak_	–0.065	–0.052
a-vO2 diff peak	0.175	0.398*
HR_peak_	–0.35	–0.167
SV_peak_	0.501**	0.445*
Q_peak_	0.453*	0.233
VE_peak_	0.534**	0.533**
RR_peak_	0.053	0.182
TV_peak_	0.331	0.375*

VO2, oxygen uptake; a-vO2 diff, arteriovenous blood oxygen concentration difference; HR, heart rate; SV, stroke volume; Q, cardiac output; VE, pulmonary ventilation; RR, respiratory rate; TV, tidal volume; *p < 0.05; **p < 0.01

## Discussion

### Peak Cardiorespiratory Variables

We observed similar VO_2peak_ values in BT and CG boys during initial testing, which was performed on the verge between childhood and adolescence. Conversely, we found that an additional year of basketball training significantly increased VO_2peak_ compared with the CG group. Some studies have suggested that training does not induce an increase in AF until a specific period during growth (Mirwald et al., 1981). In contrast, in their more recent review, Armstrong et al. (2011) concluded that VO_2peak_ can be increased by training during adolescence if the amount and intensity of practice sessions are sufficient. In support of this notion, increased AF was observed in young soccer (Chamari et al., 2005) and handball (Buchheit et al., 2009) players. However, to our knowledge, only one study has evaluated the effect of long-term basketball training on AF in similar-age boys (Vamvakoudis et al., 2007). In that study, the authors observed higher VO_2peak_ in 11.5-year-old BT boys compared with CG boys at the initial test, and the margin increased further during the 2-year follow-up; we observed a similar trend in our study.

In puberty, the increase in muscle mass is the leading cause of the increase in VO_2peak_ (Armstrong and McNarry, 2016). Therefore, absolute VO2 numbers are usually controlled for body mass or FFM, and then compared between individuals and groups. During the 1^st^ testing session of our subjects, BT boys exhibited a higher ratio-scaled VO_2peak_ for body mass (VO_2kgpeak_) than did CG boys; however, VO_2peak_ scaled for FFM (VO_2ffmpeak_) was similar in the two groups. Conversely, during the 2^nd^ testing session, both VO_2kgpeak_ and VO_2ffmpeak_ were significantly higher in BT boys. The respective variables of the CG boys remained stable between sessions, whereas both characteristics improved in the BT boys. Because body mass and FFM variables were similar between the groups (Table 1), we believe that basketball training was responsible for the additional increments observed in BT subjects, which is in line with previous data (Vamvakoudis et al., 2007).

Heart, lung, and muscle functions are additional explanatory factors for the individual differences observed in peak AF (Rowland, 2005). Basically, these systems define the oxygen pathway in the body, and they are represented in the well-known Fick equation. HRpeak and a-vO2 diff peak were reported to be independent of training in youths (Armstrong et al., 2011); therefore, SVpeak has remained at the center of interest for sports scientists. In fact, few studies have presented compelling evidence that SVpeak is the primary determinant of the increase in VO_2peak_ in children and adolescents (Obert et al., 2003; Rowland et al., 2002). Our data contradict the above-mentioned evidence. In our study, SVpeak was similar between BT and CG boys during both testing sessions (Figure 1). SVpeak ultimately depends on the heart size (Armstrong and Welsman, 2019c), and heart size generally correlates with FFM (Batterham et al., 1999); therefore, this could explain our results because the groups in our study were morphologically homogeneous. This led us to consider other components that would be able to strongly affect peak AF.

In fact, we observed a significant difference in a-vO2 diff peak between the groups during the 2^nd^ session, as well as a lower HRpeak in BT boys during the 1^st^ session. As we know, VO_2peak_ depends on HRpeak, SVpeak, and a-vO2 diff peak, and increments in any of these variables positively affect VO_2peak_. Our data suggest that one additional year of basketball training slightly improved HRpeak and a-vO2 diff peak of boys compared with their CG peers, thereby significantly increasing VO_2peak_. The possible explanation for the slight increase in HRpeak observed in the BT group may not be physiological because even earlier this variable was concluded to be similar between trained and untrained boys (Armstrong and McNarry, 2016); rather, it seems to be correlated with test performance. Although the individuals who conducted and observed the tests had sufficient experience and were motivating the boys to give their best during all runs, the possibility remains that BT boys performed their 2^nd^-year run closer to their maximal limit.

We may speculate that the wider a-vO2 diff peak observed between the groups was a consequence of training-induced peripheral adaptations related to oxygen utilization, such as muscle structure, the fiber type, enzyme activity, and capillary and mitochondrial density (Armstrong et al., 2019; Gibala et al., 2012). For example, increased capillary density increases the surface through which oxygen diffuses to muscle cells, while mitochondrial density and enzyme activity enables muscles to absorb and process oxygen at higher rates. In general terms, this means that the higher VO_2peak_ in the BT group might be related with the increased muscle quality and adaptability to the high intensity aerobic load due to constant stimuli in training sessions since the blood flow to the muscles was similar in BT and CG groups and we found no difference between Qpeak and SVpeak.

### Effect of Maturity

In basketball, more mature young players exhibit better physiological variables, perform better on-court, and are selected by coaches more often (Torres-Unda et al., 2013). Data from the above-mentioned study showed that elite players were taller and heavier and had higher FFM. Numerous studies have concluded that the maturity status has a significant effect on VO_2peak_ (Armstrong et al., 2019; Teixeira et al., 2018; TorresUnda et al., 2013). Our results are in line with those of previous studies. During the 1^st^ testing session, we observed a significant correlation between VO_2peak_ and the maturity status (*p* = 0.006). During the 2^nd^ session, this correlation was even stronger (*p* < 0.001). Absolute VO_2peak_ is expected to increase with advancing maturity because of the increase in body dimensions, mostly body mass and FFM (Carvalho et al., 2013). Although we did not calculate how cardiorespiratory values depended on morphological variables, we believe that maturity-induced morphological changes were responsible for this phenomenon.

In comparison, we did not find correlations between maturity and VO_2kgpeak_ and VO_2ffmpeak_. Regarding VO_2kgpeak_, similar findings were previously reported by other researchers (Armstrong and Welsman, 2019b). They concluded that ratio-scaled VO_2peak_ to body mass of boys remains similar or even decreases between 10 and 18 years of age. In addition, a near-linear increase in VO_2peak_ in comparison with FFM was demonstrated, suggesting that VO_2ffmpeak_ also remains stable during the pubertal years. In another study, no VO_2peak_ differences were observed between early- and late-maturing young soccer players when ratio-scaled for body mass and FFM (Teixeira et al., 2018). In the only similar study of young basketball players (Vamvakoudis et al., 2007), the maturity level of subjects was not evaluated, although the length of the study (2 years) suggests that participants experienced maturational effects. Both variables, i.e., absolute VO_2peak_ and VO_2peak_ ratio-scaled for body mass, were increased in BT boys in that study. The different results observed may be a consequence of differences in research methods, such as techniques to evaluate maturity (and/or AF), scaling variables, and sample sizes (Cunha et al., 2016). Therefore, additional studies are needed to obtain a reliable and unified way to evaluate VO_2peak_ data in children.

Regarding cardiac variables, our data showed significant correlations between SVpeak and Qpeak and the maturity level of BT boys. This is in line with the previous data reported by other authors (Armstrong and Welsman, 2002). Cardiac dimensions are the key factor for SVpeak (Vinet et al., 2003). Because one of the main functions of the cardiorespiratory system is to supply oxygen to working muscles, it is not surprising that heart size and other dimensions were associated with FFM (Batterham et al., 1999). This result also coincides with the previously mentioned finding that VO_2peak_ is largely dependent on body size. Earlier, TVpeak and VEpeak were also concluded to depend on body size during adolescence (Rowland and Cunningham, 1997), although we only found that to be true for VEpeak. HRpeak is independent of the maturity status (Rowland, 2005), and our data confirmed this statement. In addition, we also found a significant positive relationship between maturity and a-vO2 diff peak during the 2^nd^ session in BT boys. The current knowledge contradicts our findings (Armstrong and Welsman, 2002). We can only speculate that a possible explanation for these findings lies in interindividual differences and peripheral adaptation to high-intensity physical stimuli because our sample size was not sufficiently large to eliminate such a possibility. To our knowledge, no other similar studies have been conducted with BT boys of a similar age; therefore, data for comparisons are scarce.

## Limitations

Several issues should be taken into account when considering our data. First, we had no control over the training process of our BT group. Second, the activity level of children was calculated using a self-assessment questionnaire rather than electronical monitoring. Finally, participants in our control group were not fully sedentary and had some amount of leisure-time physical activity. Slight variations in the results could occur if fully sedentary children were tested. For further studies, we have a few propositions. Both genders should be tested together in this type of study, if possible. Additional subjects would allow the application of more complex, and more informative, statistical analysis methods. Lastly, longitudinal studies spanning the puberty years of children would allow differentiating between the maturity-related effects better.

## Conclusions

Basketball training improves AF of boys during early adolescence, in both absolute and ratio-scaled values and the reason for that is increased muscle’s ability to utilize oxygen rather than oxygen delivery to the working muscles. More mature young male basketball players do not present an increased AF level when considering body dimensions.
